# Draft Genome Sequences of the Kocuria subflava Type Strain KCTC 39547 and *Kocuria* sp. Strain JC486, a Newly Isolated Strain from a Wild Ass Sanctuary in Gujarat, India

**DOI:** 10.1128/mra.00535-22

**Published:** 2022-09-01

**Authors:** Jagadeeshwari Uppada, Sasikala Chintalapati, Karthika K, Venkata Ramana Chintapati

**Affiliations:** a Bacterial Discovery Laboratory, Center for Environment, Institute of Science and Technology, Jawaharlal Nehru Technological University Hyderabad, Hyderabad, India; b Department of Plant Sciences, School of Life Sciences, University of Hyderabad, Hyderabad, India; University of Southern California

## Abstract

Here, we report a 2.86-Mbp genome sequence of *Kocuria* sp. strain JC486, which was isolated from a salt marsh, and a 3.03-Mbp sequence of the type strain Kocuria subflava KCTC 39547. Prediction from their genomes indicates that both strains are nonpathogenic.

## ANNOUNCEMENT

The members of the genus *Kocuria* have been isolated from various environments and ecological niches. A few are considered potential pathogens (1) and are also able to survive in extreme environments, metal-contaminated sites, and sites with high doses of radiation, which may be exploited for biotechnological, pharmaceutical, and therapeutic applications (2, 3). In the era of drug resistance and with increasing reports of associated infections (2), it is important to predict the virulence, pathogenic potential, antimicrobial susceptibility, and biotechnological potential of new isolates from their genomes.

Here, we report the genome sequences of two strains belonging to the genus *Kocuria*, namely, Kocuria subflava KCTC 39547^T^, which was previously isolated from marine sediment from the Indian Ocean (4) and for which the genome sequence was not available, and strain JC486, which was isolated by our group from a sediment sample collected from a salt marsh located in a wild ass sanctuary in Dhrangadhra, Gujarat, India (22°98′N, 71°47′E). One gram of serially diluted (in 0.8 % [wt/vol] NaCl) sample plated (at 10^−5^ dilution) on nutrient agar (NA) (HiMedia M001) and incubated for 2 weeks at 30°C yielded yellow colonies, which were further purified by repeated streaking. Both strains were cultured in nutrient broth, and genomic DNA was extracted using a Qiagen AllPrep bacterial DNA extraction kit according to the manufacturer’s instructions. Genome sequencing was outsourced to BGI Group (Global Catalogue of Microorganisms [GCM] 10K type strain sequencing project), China (5). The read libraries with insert sizes of 300 to 400 bp were constructed by random shearing of genomic DNA with a Bioruptor ultrasonicator (Diagenode, Denville, NJ, USA) and physicochemical methods. Sequencing of the paired-end read libraries with the Illumina HiSeq 4000 system resulted in 8,112,960 and 8,062,880 raw reads from strains JC486 and KCTC 39547^T^, respectively. Raw reads of low quality from paired-end sequencing (those with consecutive bases covered by <5 reads) were discarded (6). Default parameters were selected for all of the software used in this study unless otherwise specified. SOAPdenovo v1.05 software was used to perform genome assembly (7), and annotation was carried out using the NCBI Prokaryotic Genome Annotation Pipeline (PGAP) v6.1 (8).

**FIG 1:**
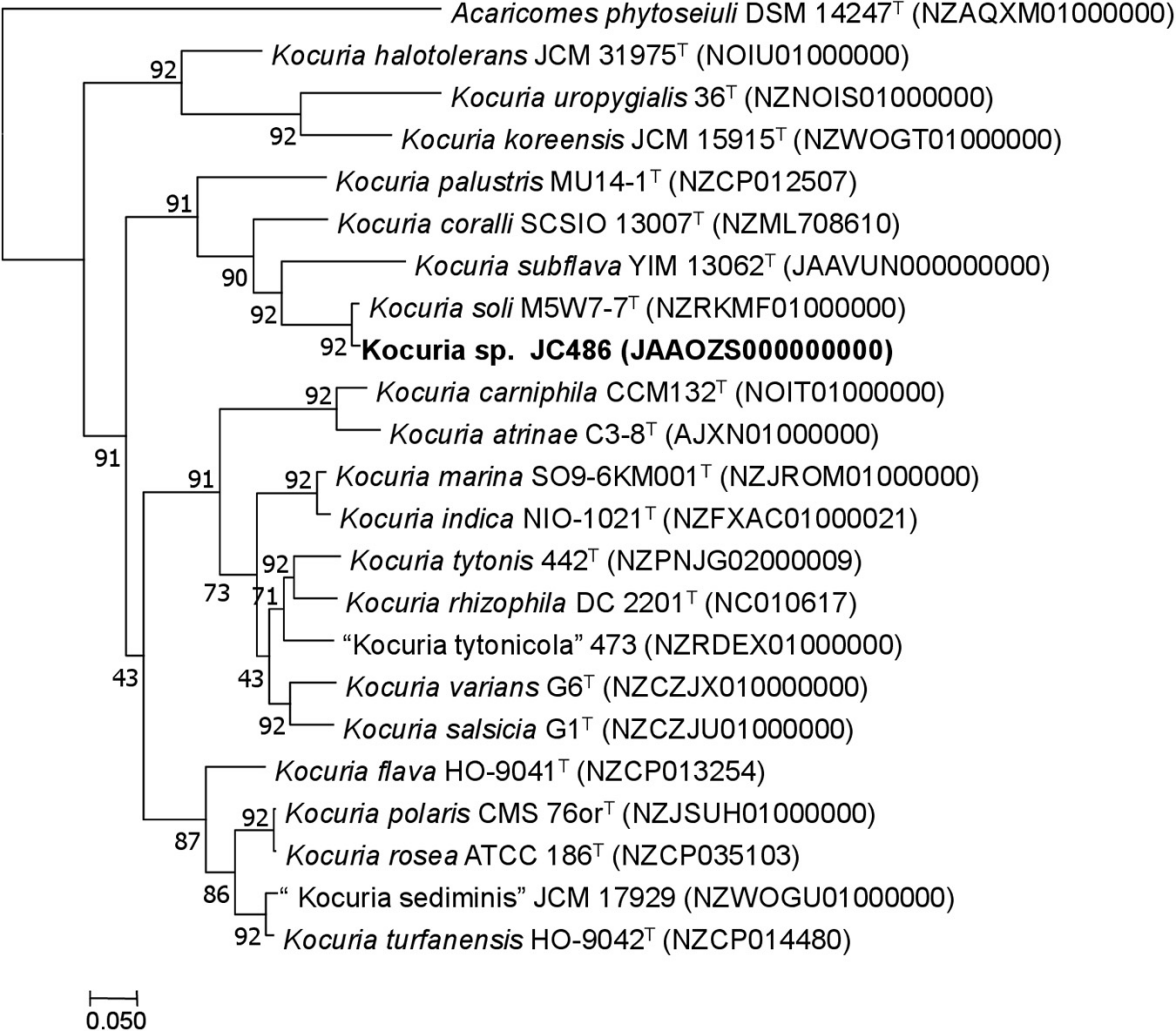
RAxML-based phylogenomic tree of members of the genus *Kocuria*. The genome sequence of Acaricomes phytoseiuli DSM 14247^T^ (GenBank accession number NZ_AQXM01000001) was taken as an outgroup. The GenBank accession numbers for the genome sequences are shown in parentheses. Bar, 0.05 nucleotide substitutions per position.

Basic Local Alignment Search Tool (BLAST) analysis using default settings for the 16S rRNA gene of strain JC468 with the EzBioCloud database (9) showed the greatest sequence similarity to Kocuria soli M5W7-7^T^ [MK163557] (99.7%) and K. subflava YIM 13062^T^ [KR610332] (97.3%), with <96.8% similarity to other members. The 92 core genes from the individual publicly available genomes belonging to the genus *Kocuria* along with strain JC486 were identified by using Prodigal (10) and hmmsearch (11), based on the Up-to-date Bacterial Core Gene (UBCG) tool (12). The extracted core genes were concatenated and aligned using MAFFT V7.0 (13), and a maximum likelihood tree was constructed using MEGA 7.0 (14). The phylogenomic tree (Fig. 1) clearly indicates the monophyletic cladding of the members of the genus *Kocuria*. The genome size of K. subflava KCTC 39547^T^ is 3.03 Mbp, with 294 contigs and an *N*_50_ value of 102,588 bp, whereas strain JC486 has a size of 2.86 Mbp, with 77 contigs and an *N*_50_ value of 108,640 bp. The G+C content and genome coverage for K. subflava KCTC 39547^T^ were 64.35 mol% and 381×, respectively, and those for strain JC486 were 67.02 mol% and 404×, respectively. PGAP annotation predicted 2,614 protein coding sequences (CDSs), 3 rRNA genes (one each of 5S, 16S, and 23S), 48 tRNA genes, and 3 noncoding RNA genes in K. subflava KCTC 39547^T^. Strain JC486 showed 2,395 protein CDSs, 4 rRNA genes (two 5S and one each of 16S and 23S), 47 tRNA genes, and 3 noncoding RNA genes. Both strains were found to be nonpathogenic when their genomes were subjected to analysis of the pathogenic nature using the online tool PathogenFinder (15). The type strain Kocuria subflava YIM 13062 is available as KCTC 38547 and CGMCC 4.7252. Strain JC486 is available as KCTC 49118, NCMR MCC 3665, and LMG 30604.

### Data availability.

The whole-genome sequences have been deposited in GenBank under the following GenBank, BioProject, and SRA accession numbers for *Kocuria* sp. strain JC486 and K. subflava KCTC 39547^T^ (= YIM13062^T^): JAAOZS000000000 and JAAVUN000000000; PRJNA602105 and PRJNA600052; and SRS6124488 and SRS6124489, respectively.
